# A Screen of the Conserved Kinome for Negative Regulators of LIN-12 Negative Regulatory Region (“NRR”)-Missense Activity in *Caenorhabditis elegans*

**DOI:** 10.1534/g3.119.400471

**Published:** 2019-09-13

**Authors:** Yuting Deng, Katherine Leisan Luo, Daniel D. Shaye, Iva Greenwald

**Affiliations:** * Dept. of Biological Sciences and; † Integrated Program in Cellular, Molecular and Biophysical Studies, Columbia University, NY 10027

**Keywords:** Notch, LIN-12, kinase, C. elegans, negative regulator, *wnk-1*, *kin-3*, *hpo-11*, *mig-15*

## Abstract

Genetic analysis of LIN-12/Notch signaling in *C. elegans* has provided many insights into human biology. Activating missense mutations in the Negative Regulatory Region (NRR) of the ectodomain of LIN-12/Notch were first described in *C. elegans*, and similar mutations in human Notch were later found to cause T-cell acute lymphoblastic leukemia (T-ALL). The ubiquitin ligase *sel-10**/Fbw7* is the prototype of a conserved negative regulator of *lin-12**/Notch* that was first defined by loss-of-function mutations that enhance *lin-12* NRR-missense activity in *C. elegans*, and then demonstrated to regulate *Notch* activity in mammalian cells and to be a *bona fide* tumor suppressor in T-ALL. Here, we report the results of an RNAi screen of 248 *C. elegans* protein kinase-encoding genes with human orthologs for enhancement of a weakly activating NRR-missense mutation of *lin-12* in the Vulval Precursor Cells. We identified, and validated, thirteen kinase genes whose loss led to increase *lin-12* activity; eleven of these genes have never been implicated previously in regulating Notch activity in any system. Depleting the activity of five kinase genes (*cdk-8*, *wnk-1*, *kin-3*, *hpo-11*, and *mig-15*) also significantly enhanced the activity of a transgene in which heterologous sequences drive expression of the untethered intracellular domain of LIN-12, suggesting that they increase the activity or stability of the signal-transducing form of LIN-12/Notch. Precedents set by other regulators of *lin-12**/Notch* defined through genetic interactions in *C. elegans* suggest that this new set of genes may include negative regulators that are functionally relevant to mammalian development and cancer.

Virtually every core component of the Notch signaling system was first identified or first linked to Notch through genetic screens in flies and worms for discrete developmental phenotypes [reviewed in (Greenwald 2012; Greenwald and Kovall 2013)], and many insights found in these invertebrate systems have been directly applicable to human biology. We now know that Notch signaling is used in a vast number of different cellular contexts for human development and for tissue homeostasis throughout adult life, and aberrant Notch activity has been implicated in many different cancers and in developmental disorders. Thus, regulating Notch signaling appropriately–in space or cell population, developmental time, strength or duration, and in response to extrinsic cues–is critical for normal development in all animals, and to avoid disease in humans.

One of the clearest associations between Notch activity and cancer has been provided by studies of T-cell acute lymphoblastic leukemia (T-ALL), for which mutations in *NOTCH1* and regulators of Notch1 activity are common drivers by bypassing important regulatory mechanisms (Chiang *et al.* 2016; Ferrando 2009). Notch is essentially a membrane-tethered transcriptional coactivator, regulated by ligand: when a ligand binds to the extracellular domain of transmembrane Notch, the intracellular domain is released by proteolytic cleavage to activate target genes [reviewed in (Greenwald and Kovall 2013)]. Missense mutations in the NRR region of the Notch1 ectodomain can bypass the need for ligand activation, and are found in about 60% of cases of T-ALL (Weng *et al.* 2004). Mutations that stabilize the untethered intracellular domain increase its activity and also promote T-ALL; such mutations delete the PEST domain of Notch1, or delete or inactivate Fbw7, the substrate-recognition subunit of a multiprotein ubiquitin ligase that targets Notch1 for degradation by a phosphodegron sequence located in the PEST domain (Ferrando 2009; Chiang *et al.* 2016).

Studies of LIN-12/Notch in *C. elegans* vulval development anticipated these key properties of T-ALL. Indeed, NRR-missense activating mutations in the ectodomain later associated with T-ALL were first observed in *C. elegans* LIN-12/Notch (Greenwald and Seydoux 1990), as was the dependence of signal transduction by these NRR-missense activated forms on the activities of γ-secretase (Levitan and Greenwald 1995) and ADAM protease (Wen *et al.* 1997). In addition, negative regulation by the conserved E3 ubiquitin ligase SEL-10/Fbw7 was also first observed via genetic interactions with mutant forms of LIN-12/Notch (Hubbard *et al.* 1997; Sundaram and Greenwald 1993). The recent finding of *VAV1* as a negative regulator of *NOTCH1* activity in promoting T-ALL (Robles-Valero *et al.* 2017) was also anticipated by the finding of *vav-1*/Vav as negative regulator of *lin-12*/Notch in *C. elegans* (Yoo and Greenwald 2005). Furthermore, components of the Cdk8 module of the transcriptional Mediator complex act as tumor suppressors and negative regulators of *NOTCH1* (Fryer *et al.* 2004; Li *et al.* 2014; Wu *et al.* 2017) and of *lin-12* in *C. elegans* (Underwood *et al.* 2017).

Here, we use a genetic interaction observed for *lin-12**/*Notch and conserved negative regulators such as *sel-10*/Fbw7 in *C. elegans* as the basis for identifying new conserved negative regulators. As protein kinases have profound and pervasive regulatory roles, we adapted the screen so as to target the 248 *C. elegans* protein kinases that had human orthologs according to OrthoList (Shaye and Greenwald 2011). This initial screen yielded thirteen protein kinases, eleven of which have never been implicated previously in regulating Notch activity in any system. Depleting the activity of five kinase genes significantly enhanced the activity of a transgene in which heterologous sequences drive expression of the untethered intracellular domain of LIN-12, suggesting that the new kinases influence the activity or stability of the signal-transducing form of LIN-12/Notch in this cell context.

## Materials and Methods

### C. elegans strains and transgenes

*C. elegans* strains used in this study are given in Table S1, and additional details about the genes and alleles may be found in WormBase (www.wormbase.org).

We used strain GS7067 [*lin-12**(**n302**)*; *nre-1**(**hd20**) **lin-15b**(**hd126**)*] for the RNAi screen of the conserved kinome and for the further analysis in Figure 2. We also used GS6392 [*lin-12**(**n302**)*; *nre-1**(**hd20**) **lin-15b**(**hd126**)*], an independently-generated strain of the same genotype as GS7067, and GS8538 [*lin-12**(+)*; *nre-1* *lin-15b*] for the analysis in Fig. S1. The genetic properties of *lin-12**(**n302**)* are described in (Greenwald and Seydoux 1990; Greenwald *et al.* 1983; Hubbard *et al.* 1997; Sundaram and Greenwald 1993) and in the text. The *nre-1**(**hd20**) **lin-15b**(**hd126**)* double-mutant was used to sensitize to feeding RNAi (Schmitz *et al.* 2007).

Strain GS8405 [*arTi43*; *arTi24*; *nre-1**(**hd20**) **lin-15b**(**hd126**)*] was used for analyzing the effect of the negative regulators of *lin-12**(**n302**)* on the activity of LIN-12(intra), the transgene-expressed untethered intracellular domain of LIN-12. Transgenes *arTi43* and *arTi24* express LIN-12(intra) in Vulval Precursor Cells (VPCs). They were derived from a plasmid in which a cDNA sequence encoding LIN-12(intra) tagged with GFP (Deng and Greenwald 2016) was placed under the control of promoter and enhancer sequences from the *lin-31* gene and the neutral 3′ untranslated region of the *unc-54* gene (based on (Tan *et al.* 1998), as in (de la Cova and Greenwald 2012) in a miniMos vector backbone (Frøkjær-Jensen *et al.* 2014). Single-copy insertions were obtained using the standard protocol (http://www.wormbuilder.org/). As expected, the single-copy insertion transgenes did not cause a highly penetrant Multivulva phenotype or display detectable GFP accumulation in VPCs in an otherwise wild-type background, individually or in combination; however, loss of *sel-10* caused a highly penetrant Multivulva phenotype and visible accumulation of GFP in the nuclei of VPCs, indicating that enhancing the activity of these transgenes may be used to assess other negative regulators. We note that we combined both transgenes in strain GS8405 because preliminary results indicated that having them together increased the sensitivity to loss of negative regulators that are not as “strong” as *sel-10* (data not shown).

### The RNA interference (RNAi) screen and validation of candidates

#### Kinome feeding RNAi library:

The set of conserved kinome genes used for screening consisted of 243 genes from OrthoList annotated as kinases based on GO terms (Shaye and Greenwald 2011) plus five (*age-1*, *atl-1*, *atm-1*, *smg-1*, and *trr-1*) that we identified manually as not being properly annotated by GO. The 248 kinases screened are shown in Table S2.

We used our recent re-assessment of worm-human orthology, OrthoList 2 (Kim *et al.* 2018), to confirm homology assignments and found that 239/248 (∼96%) of the kinase genes screened are still present in this updated compendium. Five of the kinases missing from OrthoList 2 are now described as pseudogenes (Table S2; see also www.wormbase.org, version ws272), while the remaining four (*cdk-2*, *pmk-3*, *nipi-4*, and *Y47G6A.13*) are now considered “legacy” orthologs, meaning that they were defined as orthologs by the programs used in the original OrthoList (from which our screening library was derived) but new versions of the same programs do not consider these as having human orthologs. For a Discussion of other criteria that can be used to support claims of orthology, and the treatment of “legacy” genes, see Kim *et al.*, 2018.

We assembled reagents to screen the conserved kinome by “feeding RNAi,” a procedure in which *C. elegans* is fed a collection of bacterial strains, each of which contains a clone producing double-stranded RNA directed toward an individual gene (Timmons and Fire 1998) as follows. (i) 201 kinase genes were targeted in principle by 232 clones present in the main genome-wide library used for feeding RNAi screens (Kamath and Ahringer 2003). We did not sequence-verify all of these clones, but during the course of randomly-sequencing candidates, we determined that the accuracy of the library is high, ∼90%. We replaced three incorrect clones (for the genes *daf-2*, *gck-1*, and *T01G5.1*) and augmented the coverage of two genes (*atl-1* and *pkc-1*) with additional clones. (ii) 19 kinase genes were targeted in principle by 29 clones available in another genome-wide library (Rual *et al.* 2004). However, only 14 of these clones, representing 12 genes, could be sequence-verified from this library. (iii) 35 kinase genes were not represented in either library. To complete the OrthoList kinome library, we made a clone for each of these, using genomic DNA encompassing exonic sequences, as described (Kamath and Ahringer 2003). Primers used to construct RNAi clones targeting kinases missing from existing libraries, and primers used to make non-overlapping clones to confirm candidates obtained in the initial screen, are listed in Table S3.

#### RNAi screen:

To conduct the screen, each feeding RNAi bacterial strain was fed to *C. elegans* in triplicate, with GFP and *sel-10* RNAi serving as negative and positive controls, respectively. A standard bleach/sodium hydroxide protocol (Stiernagle 2006) was used to prepare eggs from GS7067 *lin-12**(**n302**)*; *nre-1**(**hd20**) **lin-15b**(**hd126**)* hermaphrodites grown at 20°. Eggs were placed on plates containing a feeding RNAi strain and then grown at 25°. Adult hermaphrodites with three or more pseudovulvae were scored as Multivulva, with approximately 30% penetrance as a *de facto* positive result (see Figure 2). The initial screen identified 14 candidate kinase genes as potential negative regulators. All clones resulting in positive results were verified by sequencing.

#### Validating candidates:

To assess potential off-target effects, we used viable null or strong loss-of-function alleles to construct *lin-12**(**n302**)*; *kin(-)* double mutants. For all eight genes for which such alleles were available, all single mutants were non-Multivulva in a *lin-12**(+)* background but enhanced *lin-12* activity to cause a Multivulva phenotype in a *lin-12**(**n302**)* background, validating the genetic interaction inferred from RNAi. Null alleles for *kin-20*, *cdk-8*, *C24A1.3*, and *efk-1*, are homozygous viable and fertile, and enhance *lin-12**(**n302**)* activity to cause a Multivulva phenotype; null alleles of *cdk-11.1* and *hpo-11* are homozygous sterile, but homozygous segregants from heterozygous parents enhanced *lin-12**(**n302**)* activity; the strong loss of function allele *mig-15**(**rh326**)* and temperature-sensitive *par-1**(**zu310ts**)* both enhanced *lin-12**(**n302**)* activity to a Multivulva phenotype despite the non-null nature of the alleles used. We note that *C24A1.3* is now designated “*sel-15*,” for “*s*uppressor/*e*nhancer of l*in-12*” in accordance with *C. elegans* nomenclature.

When null alleles were inviable, off-target effects were evaluated by performing feeding RNAi of GS7067 [*lin-12**(**n302**)*; *nre-1**(**hd20**) **lin-15b**(**hd126**)*] in triplicate using two bacterial strains per gene, with each strain carrying one of two non-overlapping clones directed to the gene. For four genes (*csnk-1*, *wnk-1*, *gck-3*, *kin-3*), the two non-overlapping clones (Table S3) yielded Multivulva hermaphrodites, validating the inferred genetic interaction. For a fifth gene, *wee-1.3*, only one of the two clones yielded Multivulva hermaphrodites; this gene is not considered to be a validated genetic interactor and was not characterized further.

Because the background used to increase sensitivity to RNAi, *nre-1**(**hd20**) **lin-15b**(**hd126**)*, can cause a “synthetic Multivulva” phenotype when individual components of the “SynMuvA” group are depleted (Fay and Yochem 2007; Ferguson and Horvitz 1989), we also tested the ability of RNAi-depletion of each of the 14 genes to cause a Multivulva phenotype in the absence of *lin-12**(**n302**)*, *i.e.*, in a *lin-12**(+)*; *nre-1**(**hd20**) **lin-15b**(**hd126**)* background. None of the candidate *lin-12* interactors caused a synthetic Multivulva phenotype (Fig. S1), consistent with the inference that they are negative regulators of *lin-12*.

### Data availability

Strains and plasmids are available upon request. The authors affirm that all data necessary for confirming the conclusions of the article are present within the article, figures, and tables. Supplemental material available at FigShare: https://doi.org/10.25387/g3.9808256.

## Results and Discussion

### Genetic assay for negative regulators of LIN-12 Negative Regulatory Region (“NRR”)-missense activity

**Figure 1:**
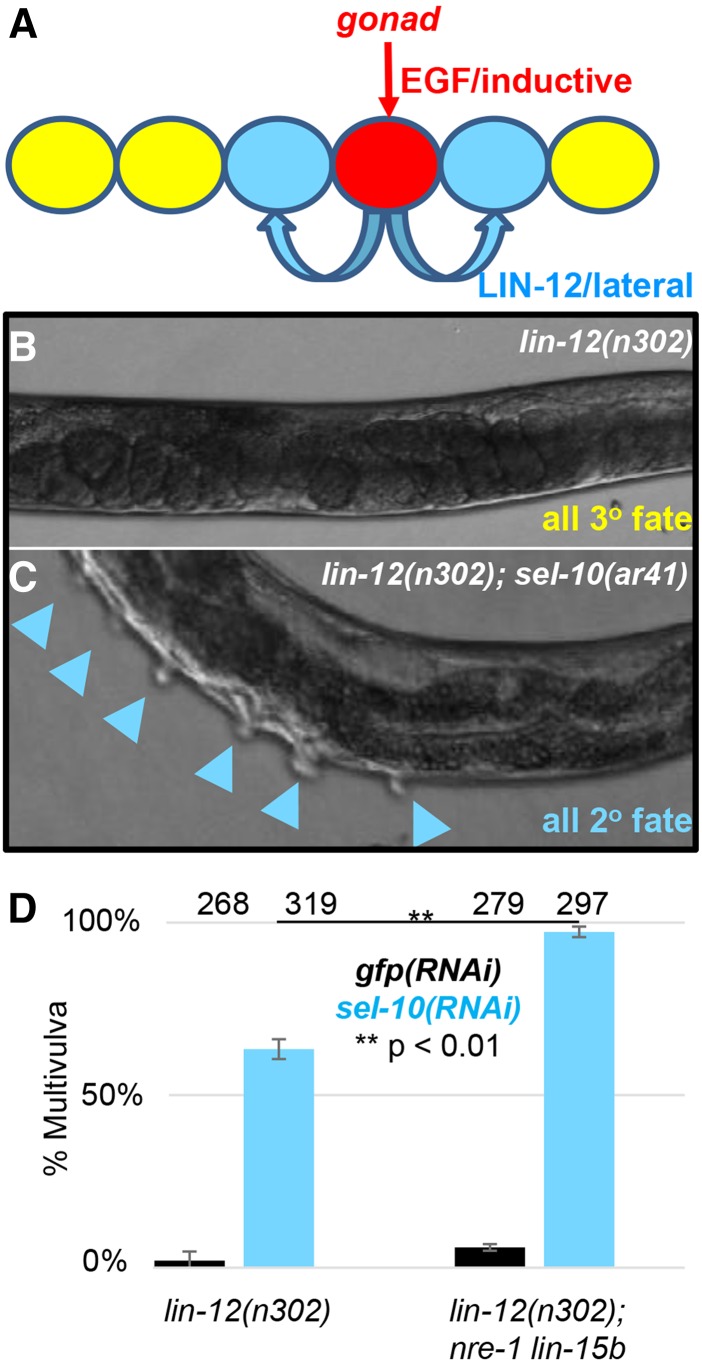
A simple phenotype-based RNAi screen for new negative regulators of LIN-12/Notch in *C. elegans*. A. Vulval Precursor Cell (VPC) fate patterning in the L3 stage of wild-type hermaphrodites. An EGF like inductive signal from the anchor cell (AC) of the gonad activates a canonical EGF like cascade in P6.p to specify the 1° vulval fate. The inductive signal also causes P6.p to express a lateral signal composed of ligands for LIN-12/Notch. The activation of LIN-12/Notch in P5.p and P7.p specifies the 2° vulval fate. B. *lin-12**(**n302**)* hermaphrodites have a relatively mild degree of constitutive *lin-12* activity. This level of activity is enough to prevent the specification of the AC, so the inductive signal is not produced; however, there is insufficient constitutive activity to cause ligand-independent specification of the VPC 2° fate (Greenwald *et al.*, 1983; Levitan and Greenwald 1995). The VPCs therefore behave like wild-type VPCs that have not received inductive signal, and all adopt the 3° fate (Sternberg 2005). C. Enhancement of *lin-12**(**n302**)* by loss of *sel-10*. Loss of a negative regulator, such as seen using a null allele of *sel-10**/*Fbw7, leads to an increase in the level of *lin-12**(**n302**)* activity, so that all VPCs can adopt the 2° fate even in the absence of an AC (Greenwald *et al.*, 1983; Sundaram and Greenwald 1993; Hubbard *et al.* 1997). D. Basis for the kinome RNAi screen. *sel-10**(RNAi)* enhances *lin-12**(**n302**)* activity, and the effectiveness of *sel-10**(RNAi)* is improved by the inclusion of *nre-1**(**hd20**) **lin-15b**(**hd126**)* to increase sensitivity to RNAi (Schmitz *et al.* 2007).

Under standard laboratory conditions, *C. elegans* develops continuously from zygote through four larval stages (L1-L4) to adulthood in 3-1/2 days at 20°. The six VPCs, named P3.p-P8.p, are born in the L1 stage and are multipotent because each has the potential to adopt one of three fates, termed “1°”, “2°”, or “3°”; they are also quiescent until the L3 stage, when cell-cell interactions specify their fates in a precise spatial pattern (Figure 1A). An EGF-like “inductive signal” from the anchor cell of the gonad activates a canonical EGFR-Ras-ERK cascade in P6.p, causing it to adopt the 1° fate. One aspect of 1° fate is to send a “lateral signal”, composed of ligands that activate LIN-12/Notch in P5.p and P7.p to specify the 2° fate. The 1° and 2° VPCs divide and generate descendants that form the vulva. The VPCs in which neither pathway is activated adopt the 3° fate, dividing to produce two daughters that fuse with the hypodermal syncytium.

**Figure 2:**
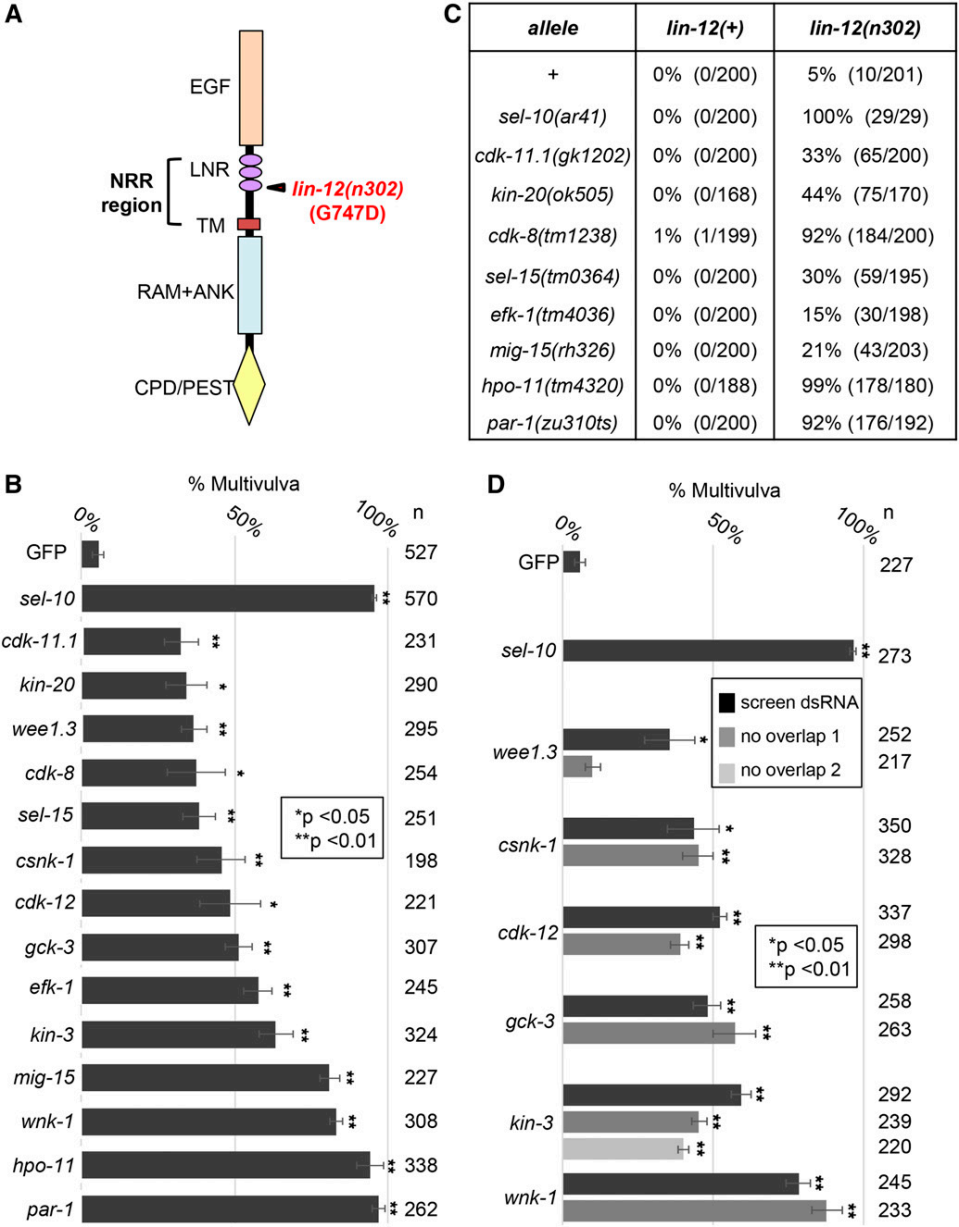
Results of the RNAi screen of the conserved kinome. A. Schematic representation of LIN-12/Notch. The domain structure characteristic of all Notch proteins is shown (reviewed in Greenwald and Kovall 2013). In the ectodomain, there is a ligand-binding region of repeated EGF-like motifs, and three LIN-12/Notch Repeat motifs, which keep the receptor inactive by blocking access to the ADAM protease cleavage site until ligand binds. The missense activating alteration in the third LNR motif associated with *lin-12**(**n302**)* is shown (Greenwald and Seydoux 1990). The NRR encompasses the LNR motif and a region called the “heterodimerization” region in mammalian Notch, which effectively extends to the transmembrane domain. In the intracellular domain, there is a “RAM+ANK” region that is critical for formation and activity of a ternary complex with a sequence-specific DNA binding protein and a bridging protein and a region containing a Cdc4 PhosphoDegron (CPD) embedded in a “PEST” protein destabilization domain that mediates SEL-10/Fbw7 binding and rapid turnover (Welcker and Clurman 2008). B. Retesting candidates from the initial RNAi screen. Fourteen initial candidates from the initial screen of the 248 C. elegans kinase genes with human orthologs were re-tested in triplicate using the initial clone present in the commercially available libraries (Kamath and Ahringer 2003; Rual *et al.* 2004) or made for this screen (Materials and Methods). *gfp(RNAi*) served as a negative control and *sel-10**(RNAi*) as a positive control, performed in parallel with kinase RNAi assessments. A Student’s T-Test with two-tailed distribution, assuming two-sample unequal variance (heteroscedastic), was used to compare the percentage of Multivulva individuals on the three RNAi plates for each kinase to the three RNAi plates for GFP in the same experiment. For an additional biological replicate testing enhancement of *lin-12**(**n302**)* as compared to *lin-12**(+)*, please see Figure S1. C. Validating candidates using conventional mutations. All eight candidates for which viable null or strong loss-of-function alleles were available were confirmed as negative regulators by constructing double mutants with *lin-12**(**n302**)* and observing enhancement of the Multivulva phenotype associated with elevated, constitutive *lin-12* activity. The null allele *sel-10**(ar41)* served as a positive control; see Materials and Methods and Table S1 for details about the nature of the alleles of kinase genes and how they were handled for this set of experiments. D. Validating candidates by additional RNAi analysis. Five of six candidates were validated as negative regulators of *lin-12* activity after a second, new RNAi clone that did not overlap with the original clone used in the initial screen was observed to enhance the Multivulva phenotype associated with elevated, constitutive *lin-12* activity; retesting the original and non-overlapping clones was performed in triplicate. *gfp(RNAi*) served as a negative control and *sel-10**(RNAi*) as a positive control, performed in parallel with kinase RNAi assessments. As in B, a Student’s T-Test with two-tailed distribution, assuming two-sample unequal variance (heteroscedastic), was used to compare the percentage of Multivulva individuals on the three RNAi plates for each kinase to the three RNAi plates for GFP in the same experiment.

NRR-missense mutations in *lin-12* have collectively been called “*lin-12**(d)*” mutations (Greenwald and Seydoux 1990; Greenwald *et al.* 1983). All *lin-12**(d)* mutations eliminate the anchor cell, but form an allelic series with respect to 2° fate: in a “weak” *lin-12**(d)* mutant, *lin-12**(**n302**)* (Figure 2A), all VPCs adopt the 3° fate, as in wild-type when the anchor cell is ablated. However, in a “strong” *lin-12**(d)* mutant, high constitutive activity causes all VPCs to adopt the 2° fate and results in a characteristic “Multivulva” phenotype, because each VPC that adopts the 2° fate generates a pseudovulva from the excess vulval tissue produced. Loss of a negative regulator such as *sel-10*/Fbw7 boosts the activity of the weak allele enough so that all VPCs now adopt the 2° fate instead of the 3° fate and become Multivulva (Figure 1B-C) (Deng and Greenwald 2016; Hubbard *et al.* 1997; Sundaram and Greenwald 1993); such enhancement is also observed by mutational reduction of proteasome activity (Macdonald *et al.* 2008). Thus, enhancing *lin-12**(**n302**)* activity to a Multivulva phenotype is a simple screen for new negative regulators of *lin-12**/Notch*.

We performed RNAi by placing eggs on Petri dishes containing a bacterial strain that expresses double-stranded RNA for an individual gene (Timmons *et al.* 2001) so that newly-hatched larvae immediately began ingesting double-stranded RNA, and examined the effects on the treated individuals directly, bypassing potential pleiotropic effects on embryonic and germline development. We optimized RNAi by using the RNAi sensitizer, *nre-1**(**hd20**) **lin-15b**(**hd126**)* (Schmitz *et al.* 2007). When *lin-12**(**n302**)*; *nre-1**(**hd20**) **lin-15b**(**hd126**)* were fed bacteria expressing double-stranded RNA for *gfp* as a negative control, few hermaphrodites had a Multivulva phenotype, whereas double-stranded RNA for *sel-10* caused a highly penetrant Multivulva phenotype (Figure 1D). The low proportion of *gfp(RNAi)* hermaphrodites displaying the Multivulva phenotype suggested it would be possible to identify enhancers that had milder effects on *lin-12* activity than *sel-10*.

### Results of the RNAi screen of the conserved kinome

In our initial screen of 248 *C. elegans* kinase genes with human orthologs (Table S2), we obtained 14 candidates based on enhancement of *lin-12**(**n302**)* activity (Figure 2B and Materials and Methods). We validated 13/14 candidates as *bona fide* negative modulators (Table 1) by testing for enhancement of *lin-12**(**n302**)* using viable null or hypomorphic alleles (Figure 2C) or, for essential genes, by observing enhancement with additional dsRNAs that did not overlap with the original dsRNA used in the screen to mitigate against off-target effects (Figure 2D). The high proportion of candidates that were validated in this way indicates that the false-positive rate for this screen was low.

**Table 1:** Human orthologs of kinase genes validated after the screen of the *C. elegans* conserved kinome^1^

*C. elegans*	Human
*cdk-8*	*CDK8*, *CDK19*
*cdk-11.1*	*CDK11A*, *CDK11B*, *[PRPF4B]*
*cdk-12*	*CDK12*, *CDK13*
*csnk-1*	*CSNK1G1-3*
*efk-1*	*EEF2K*, *[ALP1]*
*gck-3*	*OXSR1*, *STK39*
*hpo-11*	*NRBP1*, *NRBP2*
*kin-20*	*CSNK1D*, *CSNK1E*, *[CSNK1A1*, *CSNK1A1L]*
*kin-3*	*CSNK2A1-3*
*mig-15*	*MAP4K4*, *MINK1*, *NRK*, *TNIK*
*par-1*	*MARK1-4*
*wnk-1*	*WNK1-4*
*sel-15*	*TNNI3K*

^1^Orthology relationships shown here are based on OrthoList 2, a meta-analysis of six orthology prediction programs (Kim *et al.*, 2018). Human genes in parentheses were only identified by a single program, whereas other genes were identified by multiple programs. For a Discussion of other criteria that can be used to support claims of orthology, see Kim *et al.*, 2018.

The recovery of *cdk-8* and *kin-3*, the only two conserved kinase genes previously implicated as regulators of *Notch* signaling, suggests that the false-negative rate is also low. Cdk8/CDK-8 is the catalytic component of a multiprotein regulatory module of the Mediator transcription complex (Fant and Taatjes 2019). Cdk8 had been initially implicated as a negative regulator of *Notch* activity in mammals, where it is required for turnover of the Notch ternary complex (Fryer *et al.* 2004), and was then shown to act as a tumor suppressor in T-ALL (Li *et al.* 2014; Wu *et al.* 2017). *C. elegans **cdk-8* had previously been shown to act as a negative regulator of *lin-12*/Notch and to have additional roles in VPC patterning (Underwood *et al.* 2017). KIN-3 is the ortholog of the catalytic subunit of the protein kinase CK2; its regulatory subunit is KIN-10 (Litchfield 2003). Reducing the activity of *kin-3* had been shown to enhance an allele that alters the NRR of GLP-1, the other *C. elegans Notch* gene, and loss of KIN-10 had been shown to enhance *lin-12**(**n302**)* as well as the NRR mutations in *glp-1* (Wang *et al.* 2014). Our recovery of *kin-3* in our screen and characterization below, together with previous findings of Wang *et al.* (2014), strongly support the interpretation that CK2 is a negative regulator of *C. elegans Notch* proteins.

### Assaying negative regulators of lin-12(n302) for effects on the activity of the untethered LIN-12 intracellular domain

**Figure 3:**
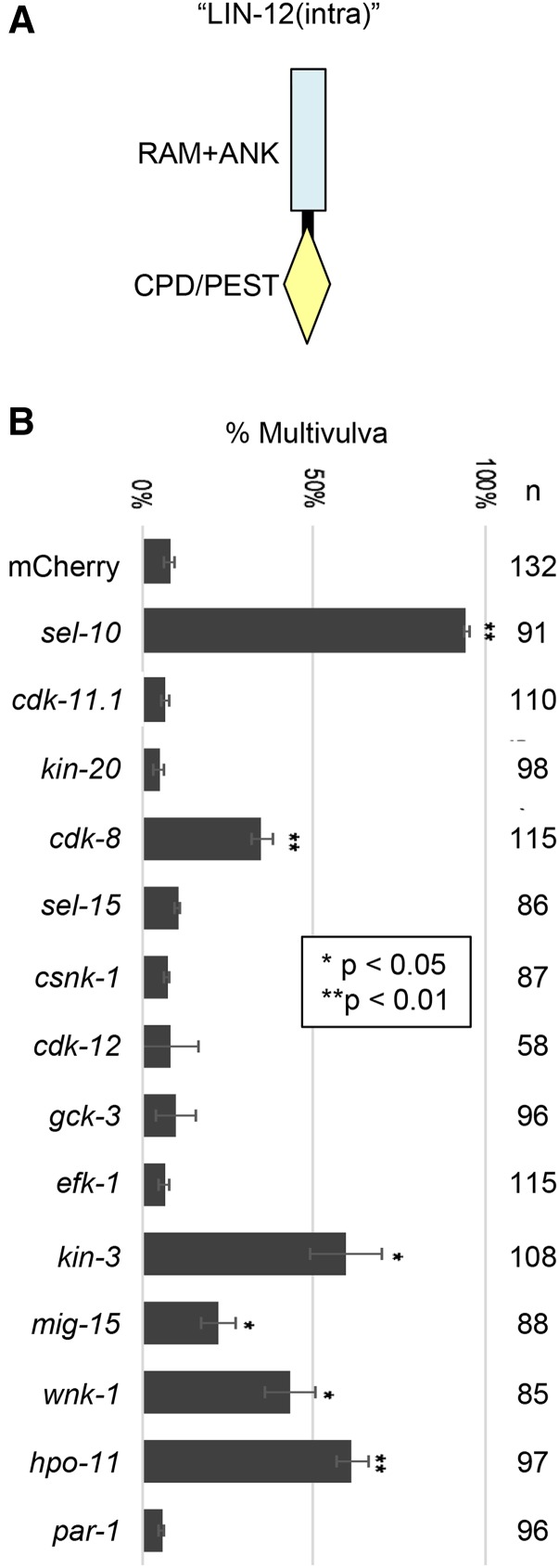
Screening new negative regulators for enhancement of LIN-12(intra) activity. A. Schematic representation of the intracellular domain of LIN-12/Notch. The untethered intracellular domain begins just after the transmembrane domain and is described in Deng and Greenwald (2016). The “RAM+ANK” and CPD/PEST regions are intact and unmutated, and are as described in Fig. 2. B. RNAi screen of new kinase genes for enhancement of LIN-12(intra) activity. GS8405, described in Materials and Methods, contains two transgenes that express LIN-12(intra)-GFP as well as the *nre-1**(**hd20**) **lin-15b**(**hd126**)* RNAi-sensitizer. *lin-12* activity is greatly enhanced by positive control *sel-10**(RNAi)* compared to negative control *mCherry(RNAi)*. Under the same conditions, five genes gave significant enhancement: *cdk-8*, *kin-3*, *mig-15*, *wnk-1* and *hpo-11*.

Expression of the untethered intracellular domain of LIN-12 [“LIN-12(intra)”] mimics the active signal-transducing form of Notch (Struhl *et al.* 1993). We assayed the effects of depleting individual kinase genes by RNAi on the phenotype of GS8405, a strain containing the same RNAi sensitizer used above and single-copy insertion transgenes that produce LIN-12(intra)-GFP in VPCs (Materials and Methods). Expression was achieved using heterologous “promoter” and 3′ UTR sequences, suggesting that any enhancement seen would be more likely to reflect enhancement at the level of the activity or stability of LIN-12(intra)-GFP protein. In this functional assay, RNAi depletion of five genes (*cdk-8*, *wnk-1*, *kin-3*, *hpo-11*, and *mig-15*) gave significant enhancement of the Multivulva phenotype (Figure 3), suggesting that they negatively regulate *lin-12**/*Notch by increasing the activity or stability of LIN-12(intra)-GFP.

We attempted to distinguish these possibilities by testing for an effect on LIN-12(intra)-GFP accumulation. As expected, *sel-10**(RNAi)* both enhanced the Multivulva phenotype of LIN-12(intra)-GFP indicative of increased *lin-12* activity and caused overt nuclear GFP accumulation (data not shown), consistent with its known function as a negative regulator of LIN-12/Notch(intra) protein stability (Gupta-Rossi *et al.* 2001; Oberg *et al.* 2001; Wu *et al.* 2001). In contrast, we did not see visible GFP accumulation in RNAi-treated animals for the kinase genes that enhanced the Multivulva phenotype (data not shown). It is difficult to interpret this negative result. It may be that these kinases inhibit LIN-12/Notch intracellular activity rather than affecting its level. Alternatively, RNAi against these genes also result in “weaker” enhancement of *lin-12* activity than *sel-10**(RNAi)*, so an associated small increase in LIN-12(intra)-GFP protein level may have been below the level of detection.

For the remaining eight kinase regulators of *lin-12* (*cdk-11.1*, *kin-20*, *sel-15*, *csnk-1*, *cdk-12*, *gck-3*, *efk-1* and *par-1*), the lack of enhancement of *lin-12**(intra)* activity despite observed enhancement of *lin-12**(**n302**)* activity would be consistent with these kinases acting at the level of the full-length transmembrane form of LIN-12(n302), *e.g.*, by altering its trafficking to increase its constitutive activity, or via regulation of the endogenous *lin-12* gene or its mRNA product.

### Concluding remarks

In our survey of the conserved kinome, we identified eleven kinase genes with genetic properties consistent with function as negative regulators of *lin-12*/Notch activity in *C. elegans* that have never been implicated in Notch activity before. We also recovered *cdk-8*, the ortholog of a known negative regulator of mammalian *Notch* (Fryer *et al.* 2004), and *kin-3*, which had previously been implicated as a negative regulator of *glp-1*/Notch (Wang *et al.* 2014). Additional functional analysis suggested that *cdk-8*, *wnk-1*, *kin-3*, *hpo-11*, and *mig-15* regulate the activity or stability of the LIN-12 intracellular domain.

Our functional screening assay of the conserved kinome was based on enhancement of a mild constitutively active form of LIN-12/Notch, and did not make any assumptions about the mechanism of action, and the list of genes provided here should serve as a starting point for further studies in *C. elegans* and other systems. We therefore note the following considerations pertinent to extending this work in the future.

First, although several of the conserved kinase genes we identified here have been studied in other processes or cell contexts in *C. elegans*–and their orthologs have been studied in diverse contexts in other organisms–such information may or may not reflect the mechanism by which they influence LIN-12/Notch activity. Indeed, it may be that the genetic interactions described here may represent an extension of the range of activities, substrates, or mechanisms of these genes.

Second, it is conceivable that the kinase genes we identified are not universal negative regulators, but are instead cell context-specific. Such context-specific effects have been observed for some genes identified in other screens for interactions with *C. elegans Notch* genes [*e.g.* (Hale *et al.* 2012; Safdar *et al.* 2016)]. VPCs are polarized epithelial cells, which may be of particular relevance in considering the question of other cell contexts in which these kinases may influence *Notch* activity.

Finally, our functional assays did not discriminate between kinases that exert a direct effect on LIN-12/Notch (*e.g.*, by phosphorylating LIN-12/Notch itself or another member of the core pathway), or an indirect effect (*e.g.*, by modulating cellular physiology), which could have pleiotropic effects that may ultimately affect signaling. However, it is important to note that even indirect effects may be functionally relevant to a cell fate decision or physiological process. For example, cell polarity and signaling have been shown to be interrelated (Niehrs *et al.* 1993) and reducing polarity genes can increase tumorigenicity associated with activated Notch in tumor models (McCaffrey *et al.* 2012).

Nevertheless, while bearing these considerations in mind for future work on these genes in *C. elegans* or their analysis in other systems, the precedents described in the Introduction suggest that among the new genes we have identified there are negative regulators that are functionally relevant to a mammalian developmental or cancer cell context. Indeed, mutations in the human orthologs of virtually all of these genes have been found in cancer patients (https://pecan.stjude.cloud/).
